# Endoscopic supraorbital eyebrow approach versus microscopic subfrontal approach for anterior cranial fossa tumor excision: A comparative study of surgical outcome and quality of life

**DOI:** 10.12669/pjms.41.13(PINS-NNOS).13379

**Published:** 2025-12

**Authors:** Rabia Saleem, Sikandar Ali, Nasruddin Ansari, Talha Sajid, Abdul Majid

**Affiliations:** 1 Rabia Saleem, MBBS, FCPS. Senior Registrar, Neurosurgery, Neurosurgery Punjab Institute of Neurosciences (PINS), Lahore, Pakistan; 2 Sikandar Ali Dehraj, MBBS, MS.Associate Professor of Neurosurgery, Neurosurgery Punjab Institute of Neurosciences (PINS), Lahore, Pakistan; 3 Nasruddin Ansari, MBBS.Resident Neurosurgery, Neurosurgery Punjab Institute of Neurosciences (PINS), Lahore, Pakistan; 4 Talha Sajid, MBBS. Resident Neurosurgery, Neurosurgery Punjab Institute of Neurosciences (PINS), Lahore, Pakistan; 5 Abdul Majid, MBBS, FCPS Professor and Head of Department, Resident Neurosurgery, Neurosurgery Punjab Institute of Neurosciences (PINS), Lahore, Pakistan

**Keywords:** Anterior, Craniotomy, Cranial Fossa, Meningioma, Neuroendoscopy, Outcome Assessment, Patient Skull Base Neoplasms, Quality of Life

## Abstract

**Objective:**

To compare surgical outcomes and quality of life following the endoscopic supraorbital eyebrow approach (ESOA) versus the microscopic subfrontal approach (MSFA) for anterior cranial fossa (ACF) tumor excision.

**Methodology:**

This retrospective comparative study analyzed 48 patients (24 per group) who underwent ACF tumor excision at the Department of Neurosurgery, Unit-III, Punjab Institute of Neurosciences between 2022 and 2024. A total of 48 patients meeting the inclusion criteria were enrolled based on convenience sampling. Patient demographics, tumor characteristics, surgical parameters, complications, and quality of life outcomes were compared between groups using appropriate statistical tests.

**Results:**

Both groups had comparable demographics, tumor sizes, and pathology distributions (predominantly meningiomas). ESOA procedures demonstrated significantly shorter operative durations (226±54 vs. 323±72 minutes, p<0.001), reduced blood loss (219.6±72 vs. 459.2±160 mL, p<0.001), and decreased hospital stays (6.6±3.2 vs. 11.6±5.1 days, p<0.001). Gross total resection rates were similar between groups (ESOA: 50.0% (12) vs. MSFA: 45.8% (11), p=0.78). While CSF leak and wound infection rates were lower in the ESOA group, only the difference in supraorbital numbness reached statistical significance (ESOA: 95.8%(23) vs. MSFA: 50.0%(12), p<0.001). Quality of life scores were significantly higher in ESOA patients (ASBQ score: 82.0±10.2 vs. 63.6±12.4, p<0.001), with better performance in social activities, reduced pain, and faster return to routine activities. Tumor recurrence rates were comparable during the 14-month median follow-up period.

**Conclusion:**

The endoscopic supraorbital approach enhances surgical efficiency, minimizes invasiveness, shortens hospital stay, and improves quality of life, offering comparable resection outcomes and serving as a viable option with careful patient selection.

## INTRODUCTION

The anterior cranial fossa (ACF) is a complex anatomical region that plays a crucial role in various neurological functions and is susceptible to a range of tumors, including meningiomas and other neoplasms. Surgical intervention in this area is often necessary to alleviate symptoms and prevent further complications. Traditionally, the surgical approaches for excising tumors in the ACF have included transcranial methods, such as the pterional and subfrontal approaches. However, the advent of minimally invasive techniques, particularly the endoscopic supraorbital approach, has emerged as a promising alternative, offering potential advantages in terms of recovery and postoperative outcomes.^1^

The endoscopic supraorbital approach (ESO) allows for direct access to the ACF with minimal brain retraction, thereby reducing the risk of neurological deficits and enhancing recovery times. This technique utilizes a keyhole approach that minimizes tissue disruption and can lead to improved cosmetic outcomes.^2^,^3^ In contrast, the subfrontal approach, while providing a broader view of the surgical field, often necessitates significant retraction of the frontal lobe, which can increase the risk of complications such as cognitive deficits and prolonged recovery.^4^ Recent studies have highlighted the efficacy of the ESO in achieving gross total resection of tumors while maintaining lower morbidity rates compared to traditional methods.^5^,^6^

Quality of life (QoL) post-surgery is a critical consideration in evaluating the success of different surgical approaches. The endoscopic techniques have been associated with shorter hospital stays and quicker return to normal activities, which significantly enhances the overall QoL for patients undergoing surgery for ACF tumors.^7^,^8^ Furthermore, the minimally invasive nature of the ESO approach is believed to contribute to less postoperative pain and reduced need for analgesics, further supporting patient recovery.^9^ In Pakistan, where healthcare resources may be limited, optimizing surgical techniques to improve patient outcomes is of paramount importance. The comparative study of the endoscopic supraorbital approach versus the microscopic subfrontal flap approach for ACF tumor excision is particularly relevant in this context. By evaluating surgical outcomes, complication rates, and QoL metrics, this study aims to provide valuable insights that could inform surgical practices in the region.

Moreover, understanding the anatomical nuances and potential complications associated with each approach is essential for neurosurgeons. The literature suggests that while the microscopic subfrontal flap approach may be advantageous for certain lesions, the ESO approach could be more beneficial for others, particularly those located laterally within the ACF.^4^ This nuanced understanding can guide surgical decision-making, ensuring that patients receive the most appropriate and effective treatment for their specific conditions. The ongoing evolution of surgical techniques for ACF tumors, particularly the comparison between the endoscopic supraorbital and microscopic subfrontal approaches, represents a significant advancement in neurosurgery. The rationale for this comparative study is not only to elucidate the surgical outcomes associated with these techniques but also to enhance the quality of life for patients undergoing these procedures in Pakistan.

## METHODOLOGY

This was a retrospective comparative study comparing the surgical outcomes and quality of life in patients undergoing anterior cranial fossa tumor excision via either the endoscopic supraorbital eye approach (ESOA) or the microscopic subfrontal approach (MSFA). The study was conducted at the Department of Neurosurgery, Unit-III, Punjab Institute of Neurosciences, Lahore, Pakistan, from 1^st^ January 2022 to 31^st^ December 2024. A total of 48 patients were identified through hospital records and included in this non-probability convenience comparative study.

### Ethical Approval:

This study was reviewed and approved by the Institutional Review Board of our Institution with reference no. 2094/IRB/PINS /Approval/2025, dated March 11, 2025.

### Inclusion Criteria:

- Patients diagnosed with anterior cranial fossa tumors who underwent surgical excision using either ESOA or MSFA.
- Age ≥18 years at the time of surgery.
- Complete medical records, including preoperative imaging, surgical details, postoperative outcomes, and follow-up data.
- Minimum follow-up period of six months post-surgery.

### Exclusion Criteria:

- Patients with incomplete medical records.
- Tumors extending beyond the anterior cranial fossa.
- Patients with previous cranial surgeries in the region of interest.
- Cases with intraoperative conversions from ESOA to Microscopic subfrontal approach.

Data was collected from patients’ medical records and Picture Archiving and Communication System (PACS). This included patient files, radiological data, operative notes, clinical notes, post-operative stay in the hospital, and post-operative clinic follow-up. A structured questionnaire was designed on Google Forms to collect data. Quality of life was assessed using the Anterior Skull Base Questionnaire (ASBQ), a validated, disease-specific assessment tool consisting of 35 questions covering six domains: Performance, Physical function, Emotional function, Pain, Specific symptoms (nasal, visual), and Social interaction.^10^ Each item is rated on a five points Likert scale (1 = poor; 5 = excellent), with total scores ranging from 35 to 175, where higher scores indicate better quality of life. The questionnaire was administered at three month post-operative follow-up visits. All ESOA procedures were performed by surgeons with at least two years of experience in endoscopic skull base surgery, while MSFA procedures were performed by neurosurgeons with similar experience in open skull base approaches.

### Statistical Analysis:

The statistical analysis employed both descriptive and inferential methods to evaluate study outcomes. For continuous variables like age and tumor size, surgery duration, blood loss, hospital stays, and quality of life (ASBQ scores) were expressed as means ± standard deviations and compared between the two groups using independent t-tests. Categorical variables, including gender, comorbidities, tumor location and type, extent of resection, postoperative complications, return to routine activities, and recurrence rates, were presented as frequencies and percentages and analyzed using chi-square tests. A p-value of less than 0.05 was considered statistically significant. Multiple comparisons were performed without adjustment (e.g., Bonferroni correction), which may increase the risk of Type-I error – future studies would incorporate appropriate multiple comparison corrections.

### Surgical Technique:

**Fig.1:**
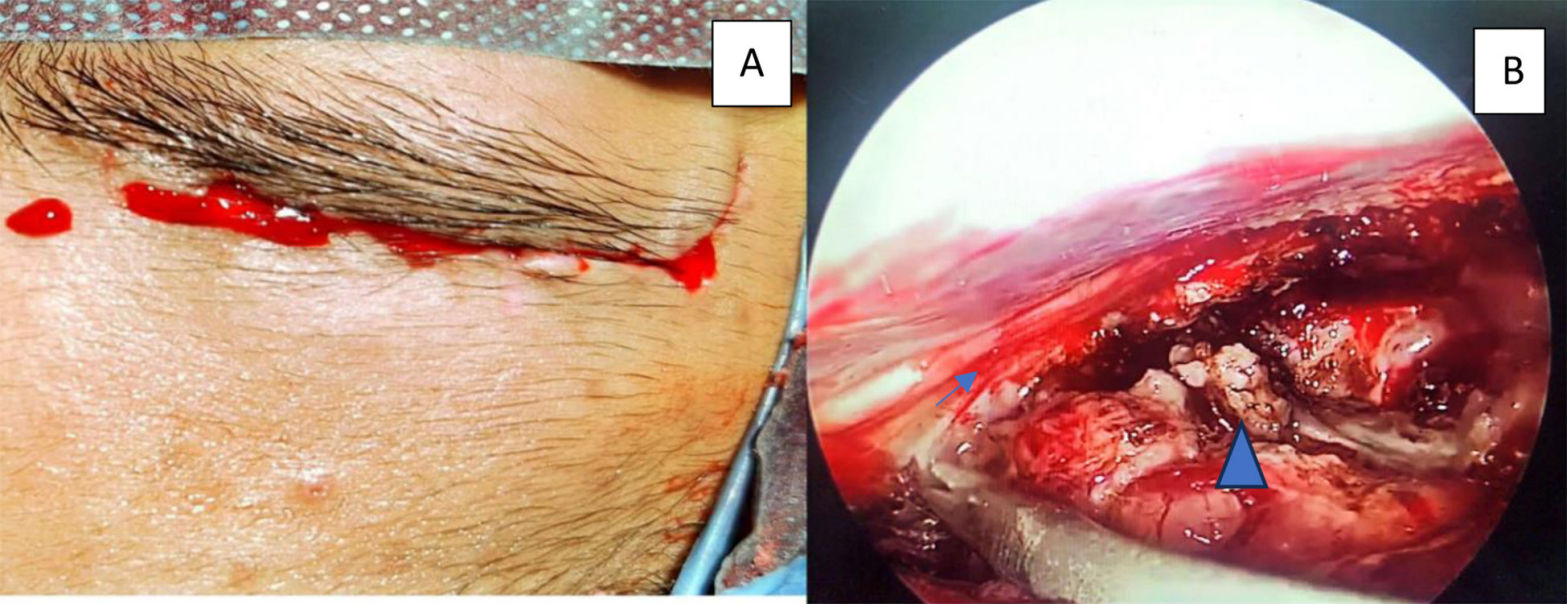
(A) shows eyebrow skin incision closure mark. (B) Arrow shows olfactory nerve and arrow head shows tumor being excised under endoscopy.

**Fig.2:**
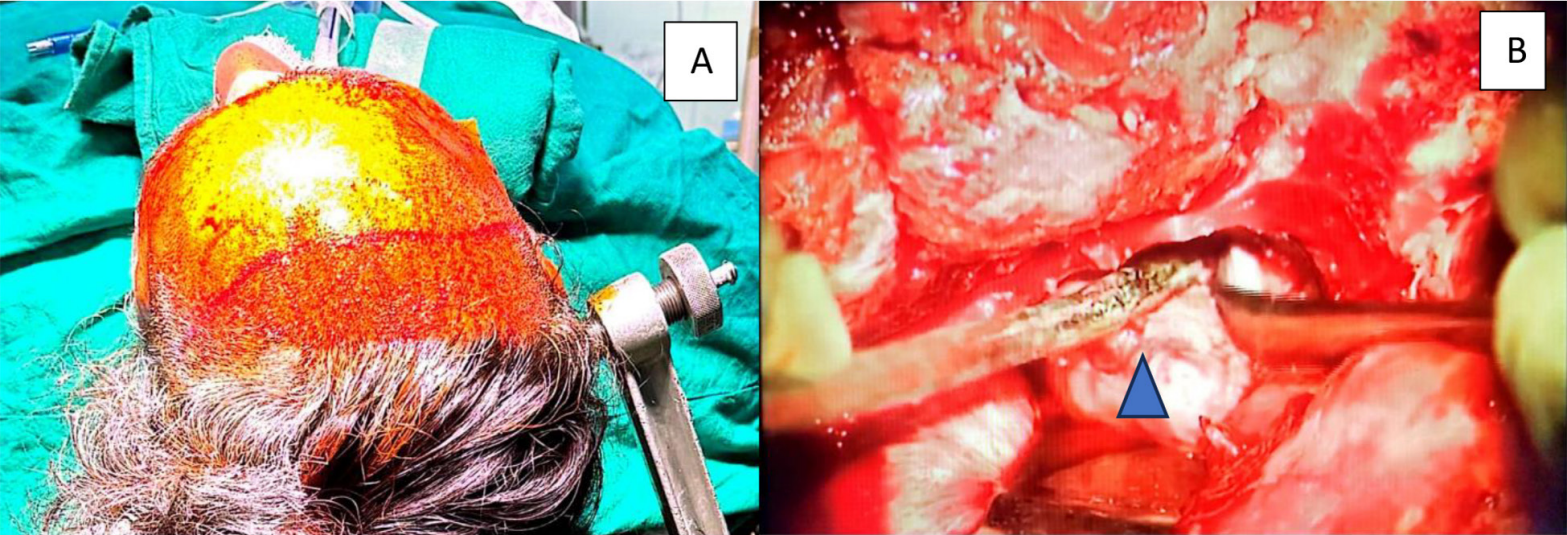
A) The patient position with the head held on the Mayfie ld frame and hairline skin incision marked. (B) Arrowhead shows the tumor being excised under the microscope.

- ***Endoscopic Supraorbital Eyebrow approach:*** The patient was positioned supine with a 20 degrees neck extension (to aid frontal lobe retraction), and his head was secured in a Mayfield head holder. The head was rotated peroperatively, taking into account the size of the lesion, and an eyebrow incision was performed. The incision goes to the lateral margin of the eye brow, sub-galeal dissection was done and scalp was retracted, frontalis muscle divides in line of incision, the curve-shaped peri-cranial base flap was raised, temporalis fascia was incised to open keyhole, the superior orbital rim was removed with eyebrow craniotomy, average dimensions are 2-3 cm wide and 1.5 to 2 cm long and bony ridges in craniotomy borders were drilled, the dura was opened in a U shape and a flap based towards the anterior skull base was fastened (retractorless approach). The CSF cisterns were opened for brain relaxation, and the endoscope was then advanced, and the procedure was completed. The pathology was addressed accordingly, as in Fig.1B. After the tumor was removed, the dura was mended. The bone flap was placed and fastened with plates, the muscle and subcutaneous layers were sutured one by one, and the skin was closed in a subcuticular way (Fig.1A).^11^
- ***Microscopic Subfrontal Approach:*** The patient was positioned supine with a 20-degree neck extension (to aid frontal lobe retraction), and his head was secured in a Mayfield head holder. The head was rotated per-operatively, taking into account the size of the lesion, and the hairline skin incision was given extending from the zygomatic root to the midline as in Fig.2A. The scalp was retracted, a musculocutaneous skin flap was raised, and the fronto-temporosphenoidal osteoplastic bone flap was raised with two burrholes. The frontal and temporal dura mater was exposed, and the orbital roof was drilled and flattened. A C-shaped dural incision with the base towards the sphenoid ridge. The CSF cisterns were opened for brain relaxation, and the microscope was then brought into the field. The tumor was excised under microscopic guidance as in Fig.2B. After the tumor was removed and hemostasis secured, the dura was closed in water-tight fashion, the bone was kept back and secured with the help of silk, the muscle and subcutaneous layers were sutured one by one, and the skin was closed with an interrupted suture technique.^12^

## RESULTS

A total of 48 patients underwent anterior cranial fossa (ACF) tumor excision, with 24 patients in the endoscopic supraorbital eyebrow approach (ESOA) group and 24 in the microscopic Sub-frontal flap approach group. The mean age was comparable between groups (ESOA: 45.3 ± 18.2 years vs. Microscopic subfrontal approach: 42.0 ± 15.8 years, p = 0.47). Sex distribution was balanced, with females constituting 58.3% (14) of the ESOA group and 45.8% (11) of the Microscopic subfrontal approach group (p = 0.40). Comorbidities were present in 66.7% (16) of ESOA patients and 62.5% (15) of Microscopic subfrontal approach patients (p = 0.77). Tumor size did not differ significantly between groups (ESOA: 2.9 ± 0.5 cm vs. Microscopic subfrontal approach: 3.1 ± 0.7 cm, p = 0.21). The most common tumor locations in both groups were the sella turcica ESOA: 33.3%(8), MFFA: 29.2%(7), and olfactory groove ESOA: 20.8%(5), Microscopic subfrontal approach: 16.7%(4). Meningiomas predominated in both cohorts, with ESOA at 62.5% (15) and Microscopic subfrontal approach at 58.3% (14) (Table-I).

**Table-I:** Demographic and Baseline Tumor Characteristics.

Variable	ESOA Group (n=24)	MSFA Group (n=24)	p-value
Age (years), mean ± SD	45.3 ± 18.2	42.0 ± 15.8	0.47
Female, n (%)	14 (58.3%)	11 (45.8%)	0.40
Comorbidities present, n (%)	16 (66.7%)	15 (62.5%)	0.77
Tumor size (cm), mean ± SD	2.9 ± 0.5	3.1 ± 0.7	0.21
***Tumor Location, n (%)***			
Sella turcica	8 (33.3%)	7 (29.2%)	-
Olfactory groove	5 (20.8%)	4 (16.7%)	-
Planum	4 (16.7%)	5 (20.8%)	-
***Tumor Type, n (%)***			
Meningioma	15 (62.5%)	14 (58.3%)	-
Craniopharyngioma	4 (16.7%)	3 (12.5%)	-
Pituitary adenoma	5 (20.8%)	7 (29.2%)	-

ESOA procedures were significantly shorter in duration (226 ± 54 minutes vs. MSFA: 323 ± 72 minutes, p < 0.001) and resulted in less intraoperative blood loss (219.6 ± 72 mL vs. MSFA: 459.2 ± 160 mL, p < 0.001). Gross total resection (≥95%) was achieved in 50% (12) of ESOA cases versus 45.8% (11) of MSFA cases (p = 0.78), while subtotal resection (75–95%) rates were comparable (41.7% (10) vs. 45.8% (11), p = 0.77). ESOA patients had shorter hospital stays (6.6 ± 3.2 days vs. MSFA: 11.6 ± 5.1 days, p < 0.001) (Table-II).

**Table-II:** Surgical Outcomes.

Variable	ESOA Group (n=24)	MSFA Group (n=24)	p-value
***Surgery duration (min),***			<0.001
Mean ± SD	226 ± 54	323 ± 72	
Median	230	305	
Interquartile Range (20^th^– 75^th^)	197-265	260-338.75	
***Blood loss (mL),***	219.6 ± 72	459.2 ± 160	<0.001
Mean ± SD	265	390	
Median	172.5-347	312-477.5	
Interquartile range (20^th^– 75^th^)			
***Extent of Resection, n (%)***			
Gross total (≥95%)	12 (50.0%)	11 (45.8%)	0.78
Subtotal (75–95%)	10 (41.7%)	11 (45.8%)	0.77
***Hospital stays (days),***			<0.001
Mean ± SD	6.6 ± 3.2	11.6 ± 5.1	
Median	6.50	10.50	
Interquartile range (20^th^– 75^th^)	4.25-8	8-14	

Postoperative cerebrospinal fluid (CSF) leak occurred less frequently in the ESOA group (25.0% (6) vs 41.7% (10), p = 0.22), and wound infection rates were lower in ESOA patients (8.3% (2) vs. 16.7% (4), p = 0.43). Supraorbital numbness was ubiquitous in the ESOA cohort (95.8% (23) vs. 50.0% (12) in MSFA, p < 0.001), while diabetes insipidus was less common in ESOA patients (16.7% (4) vs. 25.0% (6), p = 0.50). One 30-day mortality of 4.2% (1) occurred in the Microscopic subfrontal approach group (Table 3).

**Fig.3:**
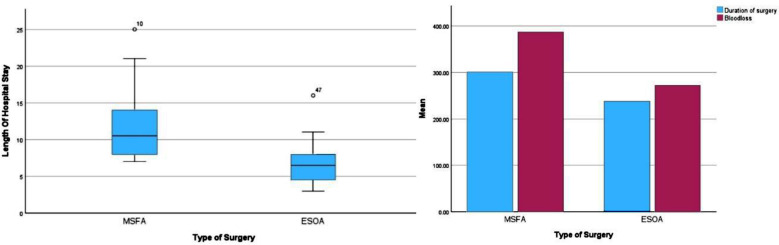
Perioperative outcomes ( Length of hospital stay, Duration of surgery, and Blood loss).

**Table-III:** Postoperative Complications.

Complication	ESOA Group (n=24)	MSFA Group (n=24)	p-value
CSF leak, n (%)	6 (25.0%)	10 (41.7%)	0.22
Wound infection, n (%)	2 (8.3%)	4 (16.7%)	0.43
Supraorbital numbness, n (%)	23 (95.8%)	12 (50.0%)	<0.001
Diabetes insipidus, n (%)	4 (16.7%)	6 (25.0%)	0.50
30-day mortality, n (%)	0 (0%)	1 (4.2%)	0.31

ESOA patients reported significantly higher postoperative quality of life (QoL) scores (mean ASBQ: 82.0 ± 10.2 vs. MSFA: 63.6 ± 12.4, p < 0.001), with subgroup analysis demonstrating better performance in social activities (p = 0.01), reduced pain (p = 0.03), and faster return to routine activities (p = 0.002) (Table-IV).

**Fig.4:**
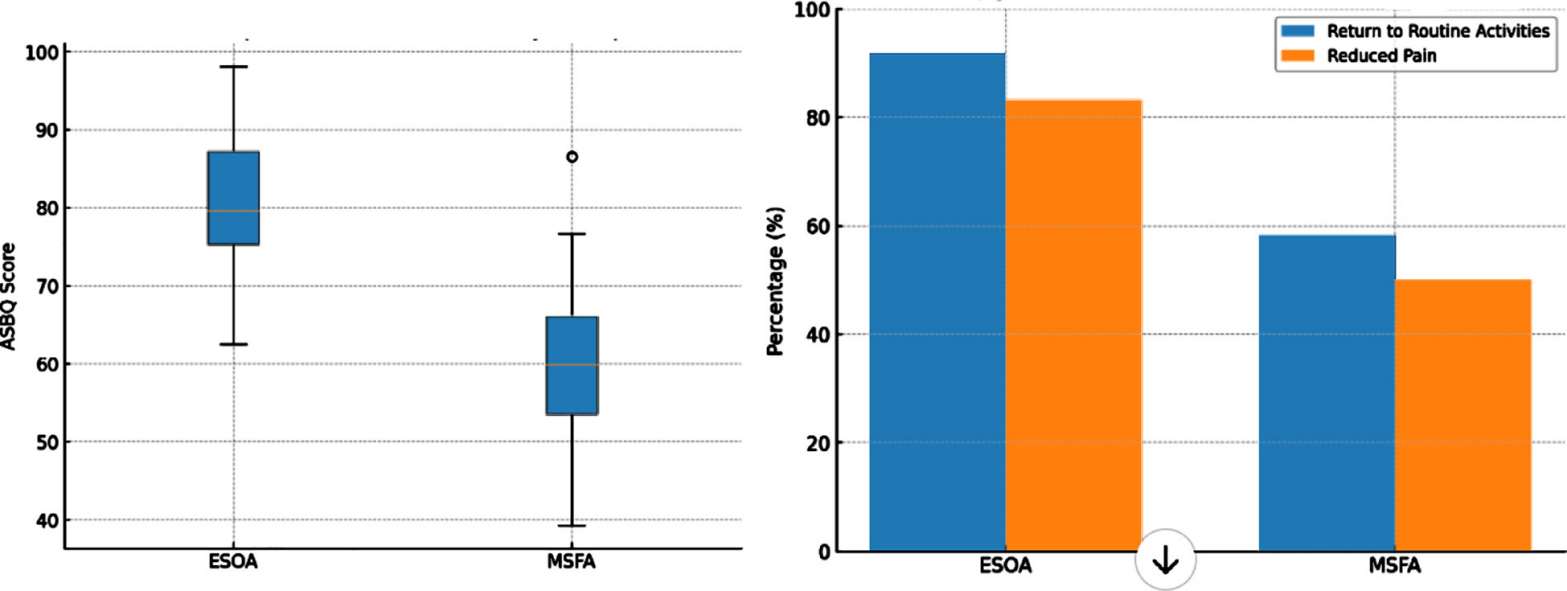
Quality of Life (QoL) outcomes (ASBQ’s score, return to routine activities, and reduced pain).

**Table-IV:** Quality of Life (QoL) Outcomes.

Variable	ESOA Group (n=24)	MSFA Group (n=24)	p-value
Postoperative ASBQ score, mean ± SD	82.0 ± 10.2	63.6 ± 12.4	<0.001
Return to routine activities, n (%)	22 (91.7%)	14 (58.3%)	0.002
Reduced pain, n (%)	20 (83.3%)	12 (50.0%)	0.03

Over a median follow-up of 14 months, tumor recurrence occurred in 8.3% (2) of ESOA and 12.5% (3) of MSFA patients (p = 0.64) (Table-V).

**Table-V:** Long-Term Outcomes.

Variable	ESOA Group (n=24)	MSFA approach Group (n=24)	p-value
Tumor recurrence, n (%)	2 (8.3%)	3 (12.5%)	0.64
Median follow-up (months)	14	14	-

## DISCUSSION

The supraorbital approach provides a reliable corridor for removing anterior skull base tumors (planum, tuberculum, and olfactory groove meningiomas), sellar and suprasellar extradural lesions (craniopharyngiomas with lateral extension), as well as intradural tumors (gliomas, metastasis) arising from the base of the skull. Incorporating endoscopy enhances the versatility of this keyhole technique and is regarded as indispensable for safe and maximal tumor excision. The management of anterior skull base lesions remains complex, and our study adds to the century-long evolution of neurosurgical strategies by comparing outcomes of minimally invasive versus traditional approaches.^13^

The ESOA offers minimally invasive access with excellent visualization of anterior cranial fossa tumors, making it suitable for patients who benefit from faster recovery, whereas the MSFA provides broader exposure and is preferred for more complex cases. ESOA is associated with shorter surgery time, less blood loss, better cosmetic outcomes, shorter hospital stay, improved quality of life, and lower healthcare costs, but it carries drawbacks such as supraorbital numbness, a steep learning curve, limited exposure for large tumors, and the need for specialized equipment.^14^

In contrast, MFFA is a familiar technique for most neurosurgeons, providing wider access and fewer cases of supraorbital numbness, though it results in longer operations, greater blood loss, larger scars, extended hospitalization, and slower recovery. Therefore, selecting the optimal approach depends on tumor characteristics, surgeon experience, and patient needs.^15^

The demographics in our study showed a relatively mature cohort, consistent with the literature recognizing that ACF tumors are more prevalent in middle-aged individuals. Additionally, the gender distribution was balanced between both the groups, with slightly females in the ESOA cohorts at 58.3%, aligning with findings from studies indicating a higher incidence of ACF tumors among females.^14^,^15^ The prevalence of comorbidities like diabetes, hypertension, hypothyroidism, and obesity was notable in our population, but comparative results were insignificant. This substantial number of patients undergoing surgery for anterior cranial fossa tumors and presenting with comorbid conditions affects surgical outcomes and recovery, influencing the choice of approach.^16^

In assessing tumor characteristics, both groups were primarily diagnosed with meningiomas, indicating a commonality in tumor types encountered in ACF surgeons. The size of the tumor and the extent of tumor resection did not exhibit a statistically significant difference, with a GTR rate of 50% for ESOA and 45.8% for MSFA, supporting findings in contemporary literature that question the extent of tumor resection achieved through various approaches.^17^ In our series, subtotal resection was only performed when GTR posed unacceptable risks of permanent neurological deficit, tumor encasement of the anterior cerebral artery, involvement of optic apparatus with calcified tumors-brain interface, and cavernous sinus invasion. Khan et al. reported GTR in MSFA for tuberculum sellae meningioma (89.6%), OGM (91.1%), followed by ESOA ( 85.2% TSM, 84.9% OGM).^18^ In a meta-analysis by Muskens et al., the overall incidence for GTR was not significantly different comparing ESOA (83%, 95% CI-76.7-88%, p value-0.74) to MSFA (incidence-85.5%, 95% CI- 83.6-87.9%,p=0.07).^19^ While some studies suggest that the ESOA may yield higher resection rates, particularly in specific tumor types such as olfactory groove meningiomas, the consensus remains nuanced and often dependent on the surgeon’s familiarity with the approach and the tumor’s anatomical context.^20^,^21^ The limited exposure of ESOA requires greater surgical skill and experience, while the wider exposure of MSFA provides better visualization at the cost of increased morbidity.

From a surgical outcome perspective, this study highlights the efficacy of the endoscopic approach exhibiting shorter surgical duration compared to MFSA group. Studies consistently demonstrate this efficacy, attributed to the enhanced visualization and maneuverability afforded by the endoscope, allowing for a more streamlined surgical procedure. The time taken for surgery was markedly less for the ESOA group (226 ± 54 minutes) compared to the MSFA group (323 ± 72 minutes), which emphasizes the efficiency of the endoscopic approach. Furthermore, intraoperative blood loss was substantially lower in the ESOA group (219.6 ± 72 mL vs. 459.2 ± 160 mL for MSFA), reinforcing findings from studies that advocate the efficacy of the endoscopic approach.^22^ Gazzeria et al., and Ndlovu et al., too, reported less blood loss in the ESOA group secondary to less tissue trauma and less need for cauterization.^23^,^24^ Post-surgical recovery times significantly favored the ESOA approach. Patients had shorter hospital stays (6.6 ± 3.2 days for ESOA vs. 11.6 ± 5.1 days for MSFA), indicating faster recovery, corroborated by studies that report similar trends. K. Mahmood et al. reported early discharge within 5 days following endoscopic excision.^11^

In terms of complications, although rates of cerebrospinal fluid (CSF) leaks and wound infections were lower in the ESOA group, the differences were not statistically significant (CSF leak: 25.0% ESOA vs. 41.7% MSFA, p=0.22 and wound infection: 8.3% ESOA vs. 16.7% MSFA, p=0.43). The pooled incidence of CSF leak in ESOA treated Tuberculum sellae meningioma was 2.11% higher than those treated by MSFA, 1.58% whereas for Olfactory groove meningioma, it was 1.61% for endoscopy vs 6.45% for microscopy.^25^ The predominance of supraorbital numbness in the ESOA cohort (95.8%) emphasizes a trade-off in sensory complications associated with this approach. K. Mahmood et al. reported complications following endoscopic excision, 2.85% of frontal numbness, 5.71% of meningitis, 1.42 % of wound dehiscence.^11^ Long-term outcomes further indicated a low recurrence rate for both techniques, ensuring that while different surgical philosophies may govern initial interventions, patient welfare must remain a focal point post-surgery.^26^

Quality of life scores, using the Abdallah Score of Quality of Life (ASBQ), were significantly higher in the ESOA group (82.0 ± 10.2) compared to MSFA (63.6 ± 12.4), demonstrating substantial improvements in postoperative recovery, pain reduction, and social activity performance.^27^ Such quality-of-life advancements resonate with findings across various neurosurgical disciplines advocating for the endoscopic approach as superior in optimizing patient outcomes.^11^ Perhaps the greatest challenge in evaluating quality of life between different surgical approaches is the fact that not all tumors are amenable to both surgical approaches. The choice of surgical approach is not only limited by the availability of surgical expertise but also by the extent and location of the tumor which may preclude either approach. Patients undergoing endoscopic approaches had significantly better quality of life compared to those undergoing open approaches at 1 year (mean change from baseline = 5.8 vs. – 1.1, *p* = 0.002).^28^

### Limitations:

The study was conducted at a single centre with a relatively small sample size, which limits the generalizability of the findings. The non-probability convenience sampling technique and retrospective nature of the analysis may introduce selection bias, and the follow-up period was relatively short, potentially underestimating the long-term recurrence rates and complications. Additionally, variations in surgeon experience and institutional practices may have influenced surgical outcomes. The absence of multivariate analysis prevents adequate control for potential confounders such as tumor size, location, and comorbidities.

## CONCLUSION

This comparative study elucidates the advantages of the Endoscopic Supraorbital Approach over the Microscopic Subfrontal Approach in terms of surgical efficiency, reduction in intraoperative blood loss, shorter hospital stays, and superior quality of life postoperatively. However, both procedures demonstrate comparable outcomes in tumor resection efficacy, highlighting that while endoscopic techniques offer numerous benefits, the selection of surgical approaches should be individualized based on patient and tumor characteristics.

### Clinical Recommendations:

The endoscopic supraorbital approach (ESOA) can be considered a safe and effective alternative to the microscopic sub-frontal flap approach for anterior cranial fossa tumor excision, particularly in well-selected patients, ESOA offers advantages in surgical efficiency, reduced morbidity, and enhanced postoperative quality of life. Clinicians should assess individual anatomical and tumor characteristics when selecting the appropriate surgical approach. Further multicenter studies with larger cohorts, randomized controlled trials with multivariate regression analysis are needed to establish the superiority of ESOA over traditional approaches and to identify optimal patient selection criteria, and long-term follow-up is recommended to validate these findings. Advanced statistical modeling such as logistic regression for complications and Cox proportional hazards modeling for recurrence analysis would provide more robust results

### Author`s Contribution:

**RS:** Concept and design of the study, critical review of the manuscript.

**SAD, NA, TS:** Data acquisition and data analysis, drafted the manuscript.

**AM:** Data interpretation, critically reviewed the manuscript and supervision.

All the authors have read and approved the final manuscript for the publication. All agreed to be accountable for all aspects of the work related to accuracy or integrity.
